# [Corrigendum] Hepa1‑6‑FLuc cell line with the stable expression of firefly luciferase retains its primary properties with promising bioluminescence imaging ability

**DOI:** 10.3892/ol.2023.13773

**Published:** 2023-03-27

**Authors:** Yasha Li, Mengnan Liu, Jiejie Cui, Ke Yang, Li Zhao, Mengjia Gong, Yi Wang, Yun He, Tongchuan He, Yang Bi

Oncol Lett 15: 6203–6210, 2018; DOI: 10.3892/ol.2018.8132

Subsequently to the publication of the above article, an interested reader drew to the authors’ attention that, in Fig. 3B on p. 6207, the data panels showing the results from the ‘Hepa1-6’ and ‘Hepa1-6-Fluc’ cell migration experiments appeared to be showing data derived from the same original source, albeit the images were rotated relative to each other.

The authors have checked their original data, and realized that the data were inadvertently selected incorrectly for the ‘Hepa1-6-Fluc’ panel in Fig. 3B. A corrected version of Fig. 3, including data from one of the repeated cell migration assay experiments in Fig. 3B, is shown on the next page. The authors are grateful to the Editor of *Oncology Letters* for granting them the opportunity to publish this corrigendum, and regret any inconvenience caused to the readership of the Journal.

## Figures and Tables

**Figure 3.**
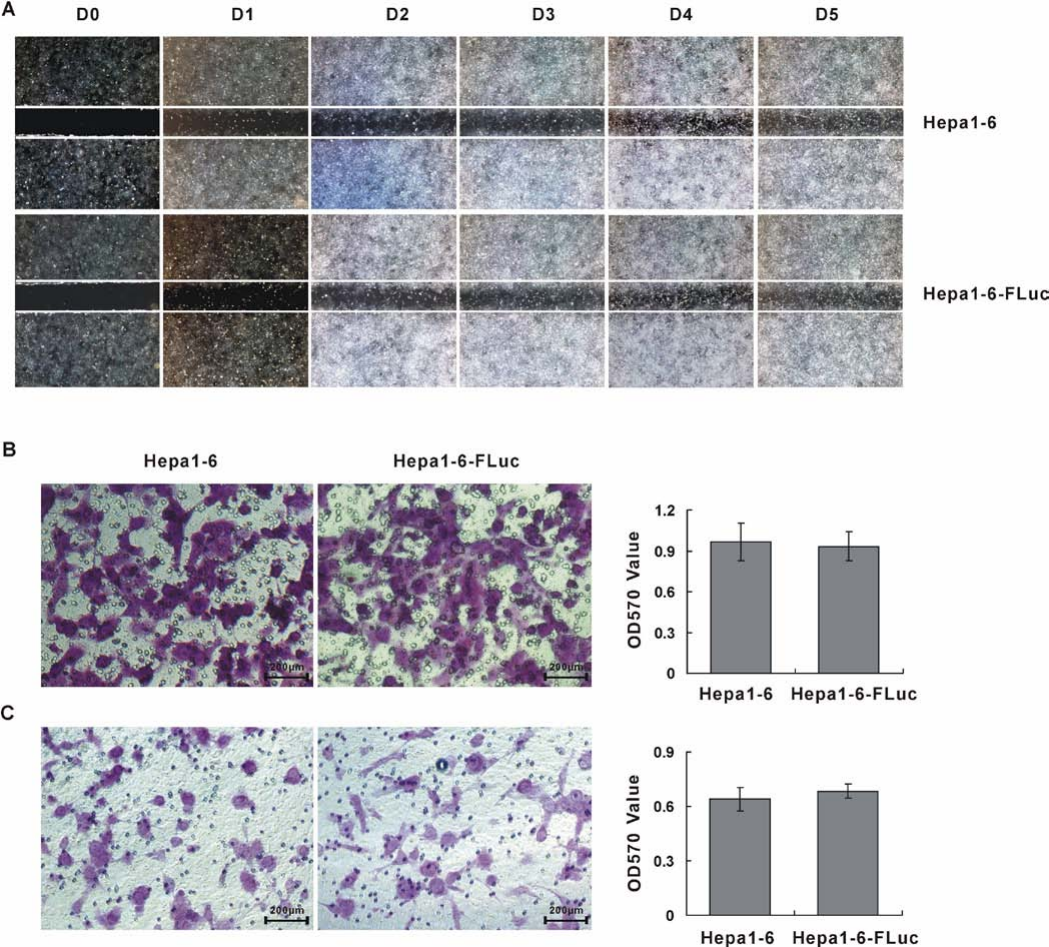
Migration and invasion ability of Hepa1-6-FLuc and Hepa1-6 cell lines. (A) Wound healing assay to detect cell migration ability. Magnification ×40. (B) Cell migration as determined by a Transwell assay. Magnification ×200; Scale bar, 200 µm. (C) Cell invasion as determined by a Transwell assay. Magnification ×200; Scale bar, 200 µm. Cells were seeded in Transwell inserts for 48 h and stained by crystal violet. FLuc, firefly luciferase; OD, optical density; D, day.

